# Neurosenescence, inflammaging and neuroinflammation in neurodegenerative disorders

**DOI:** 10.3389/fragi.2026.1756670

**Published:** 2026-03-09

**Authors:** Duraisamy Kempuraj, Prathiv Raj Ramesh Babu, Nithura Jayakumar, Mohit G. Belur, Charles H. Cohan, Arjun Sharma, Estella Sanchez-Guerrero, Tristin Anderson, Daniel Kong, Baskaran Chinnappan, Claudia Pena, Nancy G. Klimas, Theoharis C. Theoharides

**Affiliations:** 1 Center of Excellence for Neuroinflammation Research, Institute for Neuro-Immune Medicine (INIM), Dr. Kiran C. Patel College of Osteopathic Medicine, Nova Southeastern University, Fort Lauderdale, FL, United States; 2 Halmos College of Arts and Sciences, Nova Southeastern University, Fort Lauderdale, FL, United States; 3 Medical Academy for Science and Technology, Homestead, FL, United States; 4 Gateway Institute for Brain Research, Fort Lauderdale, FL, United States; 5 College of Psychology, Nova Southeastern University, Fort Lauderdale, FL, United States; 6 Miami VA Geriatric Research Education and Clinical Center (GRECC), Miami Veterans Affairs Healthcare System, Miami, FL, United States

**Keywords:** aging, Alzheimer’s disease, astrocytes, immunosenescence, inflammaging, microglia, neurosenescence, Parkinson’s disease

## Abstract

Senescence is the biological aging associated with the gradual deterioration of cells and functions of various organs over time. This irreversible process is caused by genetic, metabolic, and environmental factors, such as telomere shortening, exposure to cytotoxic substances, and accumulated cellular damage over time, although the rate of degradation can be modified by lifestyle factors. Immunosenescence specifically refers to senescent changes in the innate and adaptive immunity and is associated with low inflammation known as inflammaging. As immunosenescence implies, reduced immune function leads to impaired tissue function and an increased risk of infection and heightened susceptibility to chronic, autoimmune, and neurodegenerative disorders, such as Alzheimer’s disease (AD) in the elderly. An increase in senescent cells is common in aging, which leads to age-associated diseases. Cellular senescence may also contribute to the onset and severity of Parkinson’s disease (PD) neuropathology. Inflammaging with high levels of proinflammatory marker expression may result from changes in immune responses, chronic antigenic stimulation, and senescence-associated secretory phenotype (SASP) factors, such as increased expression of interleukin-6 (IL-6), insulin-like growth factor binding proteins (IGFBPs), transforming growth factor-beta (TGF-β) and matrix metalloproteinase-10 (MMP-10) has been reported in AD patients. The levels of the senescence marker p16INK4a and several SASP factors, such as MMP-3, IL-6, IL-1α and IL-8 are elevated along with low levels of astrocytic lamin B1 in the substantia nigra of PD. This review discusses recent developments in neurosenescence and immunosenescence in AD and PD, as well as potential senolytic therapies.

## Introduction

Aging is the natural deterioration of an organism’s physiological processes over time (Lung et al., 2021). This deterioration leads to the loss of homeostasis, which impacts health, quality, and duration of life (Rodrigues et al., 2021). An important hallmark of aging is senescence, with irreversible, gradual degradation of system functionality due to aging-related deterioration. Senescence is caused by many stimuli, including deoxyribonucleic acid (DNA) damage, activation of oncogenes, telomerase shortening, oxidative stress, mitochondrial dysfunction, and epigenetic changes. Immunosenescence is the decline of normal immune system functionality associated with aging (Ma and Fang, 2013). Cellular senescence is a similar reduction of healthy cell functioning due to multiple factors, which include altered gene expression, insufficient metabolic control, and exposure to cytotoxic compounds (Gorgoulis et al., 2019). The reduced functionality of compromised cells stops the proliferation of mitotic cells and prevents tumor formation (Lau et al., 2023). Cellular senescence is “a cell state triggered by stressful insults and certain physiological processes, characterized by a prolonged and generally irreversible cell-cycle arrest with secretory features, macromolecular damage, and altered metabolism.” (Gorgoulis et al., 2019). As such, cellular senescence is implicated in a wide spectrum of age-associated disorders (Gorgoulis et al., 2019). Because of the strong associations between senescence and disease, recent interest in therapeutic targeting senescent cells to improve healthy aging and decrease the likelihood of developing age-related diseases, known as senotherapy, has been growing rapidly. Thus, the precise detection of senescent cells is important to prospective therapeutic approaches (Gorgoulis et al., 2019).

The immune system interacts with neural, circulatory, and other systems to protect the body from internal or external pathogens (Wang et al., 2022). Immunosenescence is the age-related decrease in functionality of innate and adaptive immunity. It is linked with chronic low-grade sterile inflammation termed as inflammaging (inflammation + aging), identified by the dysregulation and high release of proinflammatory mediators; it is strongly associated with the development of multiple age-related neurodegenerative diseases (Santoro et al., 2021). Neuronal immune cells can also be impacted by inflammaging and peripheral immunosenescence, causing chronic low-grade inflammation known as neuro-inflammaging in the central nervous system (CNS) (Mohapatra et al., 2023; Franceschi et al., 2000). Senescent glial cells, such as senescent astrocytes and microglia, may contribute directly to this chronic inflammatory environment (Xia et al., 2020).

Thymic involution, naïve/memory cell imbalance, abnormal metabolism, and epigenetic changes are some characteristics of immunosenescence (Liu et al., 2023). Immunosenescence can be caused either by immune cells undergoing cellular senescence or by neighbouring senescent tissues affecting the immune system (Rodrigues et al., 2021). Genetic, hormonal, metabolic, and environmental factors, including stress, can influence the rate of senescence progression (Rodrigues et al., 2021). Immunosenescence causes high expression of inflammatory cytokines, reduced phagocytic function and vaccine response, altered surface marker expression, disrupted metabolic pathways, reduced anatomical barrier functions, reduced cytotoxicity and senescence-associated secretory phenotype (SASP) (Rodrigues et al., 2021), a group of various factors that include inflammatory cytokines/chemokines, growth factors, angiogenic factors, and matrix metalloproteinases (MMPs); these factors are called SASP or senescence messaging secretome (Gorgoulis et al., 2019). The SASP also crucially accelerates inflammation and tissue dysfunction (Bello et al., 2025). Immunosenescence and neuroinflammation are directly linked to aging and cognitive decline, as chronic neuroinflammation from aged immune cells may contribute to the pathogenesis of neurodegenerative disease (Bowirrat, 2022). Immunosenescence can cause increased morbidity and mortality in the older population, due to increased levels of proinflammatory cytokines, autoantibodies and immune cell exhaustion, creating an increased susceptibility to infection and disease (Barbe-Tuana et al., 2020; Fulop et al., 2016). Due to metabolic changes resulting from aging and senescence, some proteins cannot be effectively decomposed and thus accumulate in tissues, which also contributes to age-associated disorders (Lopez-Otin et al., 2013). In addition, microglia in aged brains exhibit hyperactive inflammasome signaling, that leads to increased oxidative stress, which can contribute to neurodegeneration (Jurcau et al., 2024).

**FIGURE 1:**
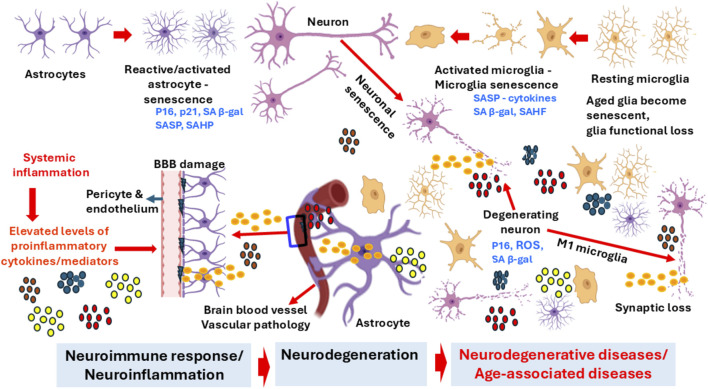
Senescence-associated brain cell alterations, neuroinflammation, and neurodegeneration. Senescent astrocytes, microglia and neurons in the aging brain. Senescent brain cells, such as neurons, astrocytes, microglia and endothelial cells, can contribute to the inflammaging, proinflammatory expression, neuronal damage, and the onset and progression of neurodegenerative diseases such as AD and PD. SASP-associated activated microglia and reactive astrocytes express many neuroinflammatory mediators that could cause chronic neuroinflammation in the aging brain. Systemic inflammation-associated proinflammatory mediators are also implicated in chronic neuroinflammation, BBB disruption, and neurodegeneration in the brain of neurodegenerative diseases. BBB, blood-brain barrier; ROS, reactive oxygen species; SASP, senescence-associated secretory phenotype; SAHF, senescence-associated heterochromatin formation.

The exact mechanism of immunosenescence is not clearly understood. Several aging-associated phenotypes contribute to immunosenescence, including SASP accumulation, chronic inflammation, reduced telomere length and telomerase activity, and metabolic alterations, which are all risk factors for age-associated disorders (Wang et al., 2022) as shown in Figure 1. Metabolic diseases of key nutrients (glucose, lipids and amino acids) in immunocytes during aging can cause dysregulation of nicotinamide adenine dinucleotide (NAD^+^) metabolism, which causes inflammation and accelerates immunosenescence. The population and proportion of senescent cells in the tissues increase with age, leading to a heightened expression of SASP inflammatory factors such as chemokines, chemokine C-X-C motif ligand (CXCL), CC chemokine ligand (CCL), growth factors and extracellular matrix proteases (Lung et al., 2021). The SASP induces senescence in an autocrine or paracrine fashion and triggers several cell signaling mechanisms, such as nuclear factor kappa β (NF-kB), mammalian target of rapamycin (mTOR) or p38 mitogen-activated protein kinase (MAPK), thereby affecting the surrounding cell microenvironment (Wang et al., 2022). However, continuous release from the SASP could lead to chronic systemic inflammation, organ injury, and inhibited immunocytes function in the aged.

The key process related to senescence is telomere shortening due to the deoxyribonucleic acid (DNA) end-replication problem in subsequent passages (Gorgoulis et al., 2019). Telomeres act as buffer caps at the ends of genetic material that protect the active, inner segments from potential damage in replication. With each successive generation, a small portion of the telomere is lost, leading to exposing chromosomal material. Although the enzyme telomerase can repair telomeres, both average telomere length and telomerase activity in lymphocytes decrease with age (Calcinotto et al., 2019). This reduced telomerase activity is generally associated with increased intracellular ROS and reduced expression of CD28 and is linked to cellular senescence pathways. Senescent CD28-T cells with shortened telomeres and low telomerase activity express fewer antiviral cytokines and high pro-inflammatory cytokines (Chou and Effros, 2013).

## Markers of aging

Senescent cells exhibit both morphological and functional changes, accompanied by altered expression of numerous markers (Table 1). Morphological alterations include cytoskeletal relocations and alterations in the cell membrane structure leading to enlargement and flattening, an irregular shape, reorganization of the nuclear lamina and reduction of lamin B1; these changes lead to altered nuclear morphology and gene expression, deactivation of proliferation-associated genes, increased cellular stress pathways, and increased expression of senescence-associated β-galactosidase (Behfar et al., 2022). Useful biomarkers should not only forecast individual age-specific morbidity and age-associated abnormalities, more precisely than chronological age alone, but also serve as indicators of biological risk factors (Lung et al., 2021). Several “hallmarks” and “pillars” of aging have previously been reported in the literature, such as “genomic instability, telomere attrition, epigenetic changes, loss of proteostasis, dysregulated nutrient sensing, mitochondrial disorder, cell senescence, stem cell exhaustion and abnormal intercellular communication” (Lung et al., 2021; Calcinotto et al., 2019) (Table 1). Recently, senescence gene sets have been developed to further study these processes (Saul et al., 2022). One paper reported elevated blood levels of “alkaline phosphatase, cholesterol, clotting factors VII and XIII, D-dimer, ferritin, fibrinogen, postprandial glucose, parathormone, interleukin-6 (IL-6), vitamins, noradrenaline, parathyroid hormone, prostate specific antigen, triglycerides, uric acid, and HbA1c” in the elderly. They also reported decreased levels of “calcium, zinc, creatine kinase, estimated glomerular filtration rate (eGFR), dehydroepiandrosterone, testosterone oestrogen, growth hormone, insulin-like growth factor-1 (IGF-1), IL-1, phosphor, selenium, thiamine, Χ-tocopherol (vitamin E), vitamins B6 and B12, vitamin C, vitamin D, and alanine aminotransferase” in the elderly (Lung et al., 2021). Inflammatory markers, such as nuclear factor-kappa β (NF-kB), are linked with enhanced aging, alopecia, kyphosis, osteoporosis, CNS alterations, elevated cellular senescence, reduced regenerative capacity, and reduced lifespan in aged mice (Rodrigues et al., 2021). Recently, senescence gene sets have been developed to further study these processes (Saul et al., 2022). Soluble urokinase-type plasminogen activator receptor (suPAR) is a biomarker for biological brain aging, as high suPAR levels are associated with older individuals (Rasmussen et al., 2021). Recent studies reported the importance of IL-11 in aging (Airapetov et al., 2023; Cook, 2023; Nishina et al., 2021; Zhu et al., 2025; Seyedsadr et al., 2023). Recently human cellular senescence real-time polymerase chain reaction array is available to detect senescence-associated gene expression changes in models of replicative senescence, oncogene-induced senescence (OIS), therapy-induced senescence (TIS), and age-related tissue dysfunction.

**TABLE 1:** Age-associated markers.

Marker (blood/serum/plasma/CSF/cells/tissues)	Cells/cell source/expression	Expression levels in diseases	Diseases/conditions associated	References
SASP - cytokines IL-1β, IL-6, IL-8, GDF15, FAS, OPN, TNFR1, ACTIVIN A, CCL3, IL-15, CST3, IGFBP7	Senescent cells/immune cells	High secretion	MCI	St Sauver et al. (2023), Muthamil et al. (2024), Rim et al. (2024), Ma et al. (2025), Gorgoulis et al. (2019), Banarjee and Basisty (2025)
Cell cycle arrest (G0-G1 phase)	Senescent cells	Cell proliferation stops	MCI	SanMartin et al. (2023), Salech et al. (2022)
β-galactosidase	PBMC	Altered	MCI, AD	Wagner and Wagner (2022), Gorgoulis et al. (2019), Mohamad Kamal et al. (2020), de Mera-Rodriguez et al. (2021)
Lamin B1	Nuclear lamina	Low	Senescence	Hernandez-Segura et al. (2018), Dreesen et al. (2013)
MPO, MMP-1, MMp7	Plasma	Altered	MCI	Mielke et al. (2025)
Transcriptomic and Multi-Omics	Biomarkers	DNA damage, cell cycle and inflammatory genes	AD	Mahmud et al. (2024)
Telomere attrition	Blood, brain tissue, senescent cells	Shortening	Cellular senescence, genomic instability, compromised regeneration and disease worsening	Asghar et al. (2022), Wang et al. (2024)
Reduced NK cell-activating cytokines IL-2, IFN-α and IFN-γ	Immune cells	Reduced	Aging	Wang et al. (2022)
DNA damage and chromatin changes, γH2AX	Senescent cells	Damage/Changes/genomic instability	Aging	Hernandez-Segura et al. (2018), Mohamad Kamal et al. (2020)
CD4^+^CD27^−^CD57^+^ T cells, late memory B cells	Senescent immune subset	Increased	Progression of ALS	Yildiz et al. (2023)
CD57 - CD28	T cell senescence markers	Altered	PD	Kouli et al. (2021)
Autoantibodies, peripheral B cells decreased, but increased proinflammatory B cells and ABCs	B-cells	Increased proinflammatory and B cells and ABCs	Aging, Autoimmune diseases	Wang et al. (2022), Valentino et al. (2024)
PD-1	Senescent cells	​	Aging	Salminen (2024)
PD-L1	Senescent cells	Increased	Aging	Salminen (2024)
Gene expression in PBMCs	​	High	Neurodegeneration	Grandke et al. (2025)
PPIA, HSC70, hnRNPA2B1, TDP-43	Protein markers	Altered in PBMCs	ALS	Pansarasa et al. (2022)
TNF-α	​	Increase	Age-associated diseases	Michaud et al. (2013)
GFAP & GFAP breakdown products (BDP)	Astrocytes	Increased	Increases in ageing, astrogliosis, and neurodegenerative diseases	Sharma et al. (2022), Thangavel et al. (2024), Ahmed et al. (2017), Kim and Kim (2024)
P16 INK4A, p21, p53	​	High	Aging	Gorgoulis et al. (2019), Kudlova et al. (2022), Salech et al. (2022), SanMartin et al. (2023)
NfL	Neurons	Middle-aged and older individuals, Aging	Ageing, predicting mortality, COVID-19, TBI, stroke, AD, MCI, axonal injury	Nguyen et al. (2022), Palmer et al. (2025)
pTau181, pTau231, tTau	Neurons	High	MCI, AD can distinguish AD from other dementias. More in AD than in cognitively unimpaired patients or non-AD dementias, in NFTs	Teunissen et al. (2022), Palmer et al. (2025)
Mitochondrial dysfunction	Senescent cells	Altered	Aging, PD, AD	Asghar et al. (2022), Ma et al. (2025), D'Alessandro et al. (2025)
Reduced CD45RA, CD27, CD62L, CCR7 and increased CD45RO, CD57. High SA-β-Galactosidase activity, p16NK4a, macroH2A, dysfunctional telomeres	Cytotoxic T cells	Reduced cytotoxicity, increased disease risk	Aging	Calcinotto et al. (2019), Rodrigues et al. (2021), Wang et al. (2022)
Ki67	Nuclear protein	Loss	Aging	Kudlova et al. (2022), Zhu et al. (2024)
TNF-α, IFN-γ, perforin, granzymes	Natural killer cells, NK cells, cytotoxic lymphocytes	​	COVID-19, cancer, virus-infected cells, Gulf War Illness	Esen and Turner (2025), Richardson et al. (2024)
Aβ 1-42, Aβ42/Aβ40 ratio	Neuron	High	Elevated with age, Preclinical AD, predementia, AD, neurotrauma	Teunissen et al. (2022)
pTau181, pTau231, tTau	Neuron	High	MCI, AD, can distinguish AD from other dementias. More in AD than in cognitively unimpaired patients or non-AD dementias, in NFTs	Teunissen et al. (2022)
α-synuclein aggregation	Dopaminergic neurons	High	PD	Ma et al. (2025), Thangavel et al. (2024), Palmer et al. (2025)
Claudin-5, occludin, ZO-1 - tight junction proteins	Neurovascular unit-endothelial cells	High	BBB dysfunction in healthy ageing is a typical process, including cognitive impairment, systemic inflammation, LPS	Sharma et al. (2022)
CCL11 (eotaxin)	Eosinophil/mast cells	High	Neurodegenerative disorders, eosinophil recruitment, allergic responses/asthma	Brito-de-Sousa et al. (2022)
PDGFRβ	Pericytes	High	BBB disruption in healthy ageing is a typical process, and cognitive impairment	Sharma et al. (2022)
Aquaporin-4	Astrocyte end-feet/neurovascular unit	Low	Reducing with ageing, BBB disorders, AD, MS, systemic inflammation, LPS	Sharma et al. (2022)
GFAP & GFAP breakdown products (BDP)	Astrocytes	Increased	Increases in ageing, astrogliosis, and neurodegenerative diseases	Sharma et al. (2022), Thangavel et al. (2024), Ahmed et al. (2017), Kim and Kim (2024)
VCAM-1	Endothelial cells	Aging, chronic inflammation	Ageing, peripheral chronic inflammation	Sharma et al. (2022)
ICAM-1	Endothelial cells	Aging, chronic inflammation	Aging, peripheral chronic inflammation	Sharma et al. (2022)
MMP3	neuronal injury	High	Aging	Sharma et al. (2022)
VEGF	​	High	Inflammation	Sharma et al. (2022), Rodrigues et al. (2021)
CXCL-8/(IL-8)	Immune cells	High	Inflammation, chemotaxis of neutrophils, arthritis	Brito-de-Sousa et al. (2022), Rodrigues et al. (2021)
IL-6	Immune cells	High	Aging-cognition disorder	Brito-de-Sousa et al. (2022), Rodrigues et al. (2021), Michaud et al. (2013)
IL-11	Astrocytes, monocytes, macrophages, endothelial cells, damaged cells	High	Aging	Widjaja et al. (2024), Nishina et al. (2021), Airapetov et al. (2023), Seyedsadr et al. (2023)
IL-1β	Immune cells	High	Aging-cognition disorder	Brito-de-Sousa et al. (2022), Rodrigues et al. (2021), Michaud et al. (2013)
ROS	Immune cells	High	Aging	Sharma et al. (2022)
CK	Muscle	High	Muscle injury, muscle damage, neuromuscular disorders, chronic pain	Aleksovska et al. (2025)
GH, testosterone, estrogen, cortisol	Endocrine cells	Altered	Aging, biomarkers of endocrine function	Lara et al. (2015)
IL-7	Stromal cells	High	Immunosenescence	Rodrigues et al. (2021)
IL-15	Immune cells	High	Immunosenescence, aging	Rodrigues et al. (2021)
CXCL1 (GROα)	Aged cells	High	Immunosenescence, tissue damage	Rodrigues et al. (2021)
MCP2	Immune cells/Senescent cells	High	Immunosenescence	Rodrigues et al. (2021)
IL-17	Th17	Aging	Autoimmune diseases	Sola et al. (2023)
Old cells	Telomere shortening, SASP expression	Altered	General cell aging	Lung et al. (2021)
CD4^+^, CD28^−^, CD8^+^CD28−IFN-γ, TNF-α	T cells –	Altered	Immune response	Lung et al. (2021)

Abbreviations: Aβ, beta amyloid; ABCs, age-associated B cells; AD, Alzheimer’s disease; aMCI, amnestic mild cognitive impairment; ALS, amyotrophic lateral sclerosis; BBB, blood-brain barrier, CCL11, C-C chemokine ligand 11; CK, creatine kinase; CXCL-8, C-X-C motif chemokine ligand 8; GFAP, glial fibrillary acidic protein; ICAM-1, intercellular adhesion molecule; IL, interleukin; IFN-γ, interferon gamma; GWI, gulf war illness; LPS, lipopolysaccharide; MMP-3, matrix metalloproteinase 3; MCI, mild cognitive impairment; MS, multiple sclerosis; MPO, myeloperoxidase (MPO); NfL, neurofilament light; NFT, neurofibrillary tangle; NK cells, natural killer cells; PD-1, programmed cell death protein 1; PDGFRβ, platelet-derived growth factor receptor beta; PBMCs, peripheral blood mononuclear cells; pTau, phosphorylated tau; SA-β-gal, senescence-associated β-galactosidase; TBI, traumatic brain injury; TNF-α, tumor necrosis factor alpha; RONS, reactive oxygen and nitrogen species; tTau, total tau, ROS, reactive oxygen species; VCAM-1, vascular cell adhesion molecule 1; VEGF, vascular endothelial growth factor; ZO, zonula occludens.

## Neuronal and glial senescence

The aging brain shows a low-level chronic inflammation, likely contributed to by cellular senescence (Behfar et al., 2022). Possible senescence-related brain cell alterations, neuroinflammation, and neurodegeneration are shown in Figure 1. However, studies related to neuronal senescence are currently limited (Behfar et al., 2022). Post-mitotic cells that no longer divide can become senescent in response to stress (Sapieha and Mallette, 2018). As neuronal cells are post-mitotic cells, senescence must depend on mechanisms distinct from proliferation arrest (Behfar et al., 2022). Elevated expression of p16 and transcription factor GATA4 (a SASP initiator) were shown in neurons in the aged (Kang et al., 2015). Astrocytes in aged brains express more p16 compared with those in younger brains (Liu et al., 2023).

Another study suggested that p19 is a significant contributor to senescence in postmortem human brains (Dehkordi et al., 2021). With aging, neurons display morphological and functional changes, accompanied by changes in proteostasis, redox balance, and Ca2^+^ dynamics; however, it is unclear whether these changes are indicative of senescence (Ma et al., 2025).

Microglia are non-neuronal support cells of mesenchymal origin that function as resident macrophages in the CNS. They constantly sample the extracellular space by extending several ramified processes. Microglial activation typically protects the brain. However, senescence in microglia presents with dystrophy, reduced motility, abnormal signaling, impaired phagocytosis, and impaired proteostasis-associated phenotypes that are termed the “hallmarks of microglia aging” (Behfar et al., 2022). These senescent microglia are identified in several brain regions during aging, making the brain increasingly vulnerable to injuries and infections.

Astrocytes are the largest proportion of glial cells in the brain, supporting neuronal functions and regulating the integrity of the blood-brain barrier (BBB) (Kempuraj D et al., 2024; Cohen et al., 2024; Kempuraj et al., 2020). Astrocytic dysfunctions are observed in normal aging and age-dependent neurodegenerative disorders. Several external factors, specifically oxidative stress, can induce astrocyte senescence and increase the expression of senescence-associated p16, p21, p53, and SASP *in vitro* astrocyte culture (Bitto et al., 2010). A significant decrease in lamin B1 in astrocytes of the hippocampus has also been reported in post-mortem brain tissue of aged individuals (Matias et al., 2022).

## Neurosenescence and immunosenescence in brain disorders

The brain is composed of neurons, astrocytes, microglia, and oligodendrocytes, all of which are susceptible to senescence. Immunocytes can only proliferate up to a fixed number of divisions before transforming into senescent cells, at which point they demonstrate a reduction in proliferation ability (Lau et al., 2023). This reduction can progress to a permanent halting of cell growth, which has been associated with Multiple Sclerosis (MS), Alzheimer’s disease (AD), Parkinson’s disease (PD), and epilepsy (Bello et al., 2025). Exposure to oxidative compounds, such as reactive oxygen and nitrogen species (RONS), can irreversibly damage proteins and is another factor that contributes to the pathogenesis of senescence. The extent to which age-related cellular senescence affects brain health and contributes to neuropathogenesis in PD is not yet clearly known (Ma et al., 2025). Microglia, the resident immunocyte of the brain, phagocytose senescent cells in the brain (Lau et al., 2023). Astrocytes release trophic factors, including IL-3, which help reduce the microglial M1 proinflammatory phenotype (McAlpine et al., 2021). Inhibition of autophagy can cause neuronal and glial senescence in aging brains and in response to neurodegeneration-associated stresses (Palmer et al., 2025).

In immunosenescence, mast cell release decreases, whereas mast cell degranulation increases; phagocytosis decreases with increased inflammatory mediator release from macrophages; decreased cytotoxicity of natural killer cells; and observation of decreased chemotaxis of neutrophils (Grizzi et al., 2013). A recent study indicated that aging increases the number of mast cells in many tissues, with an increase in the overall histamine pool (Kamphuis et al., 2025). Another recent study reported that senescence affects mast cell function and is linked to inflammaging (Ibarra-Sanchez et al., 2025). Mast cells are found in the CNS and may play a role in neuroinflammation (Theoharides et al., 2023).

A paper reported that the aging immune system contributes to the morbidity and mortality of the aged (Yousefzadeh et al., 2021). Increased senescence and damage in non-lymphoid organs indicate that senescent and aged immune cells can contribute to systemic aging, representing an important therapeutic target to extend healthy ageing (Yousefzadeh et al., 2021). This study also demonstrated that treatment of *Vav-iCre* ^*+/−*^ *;Ercc1* ^*−/fl*^ mice with rapamycin reduced markers of senescence (p16 and p21 in CD3^+^ peripheral T cells) in immune cells and improved immune function and reduced serum levels of CCL2/MCP-1 and TNF. Immune cells are vulnerable to endogenous DNA damage, that can cause cell death or senescence. The mechanism behind parenchymal damage appears to be a combination of cell-autonomous (loss-of-function) and cell-non-autonomous (gain-of-function, for example, SASP).

Suppression of target of rapamycin complex 1 (TORC1) by rapamycin enhances healthy aging. Inhibition of S6 kinase (S6K) lowers age-related inflammation and increases lifespan through the endolysosomal system. A recent study demonstrated that activation of S6K in *Drosophila* blocked extension of lifespan by rapamycin, increased multilamellar lysosomes and blocked age-associated increased activation of the NF-κB pathway, and reduced inflammaging (Zhang et al., 2024). Additionally, this study also reported that rapamycin elevated the levels of Syntaxin 12/13 in the mouse liver and prevented the age-associated increase in NF-κB signaling (Zhang et al., 2024).

Aging is an important risk factor for cancer. Aging of the immune system, regardless of the age of the stroma and tumor, accelerates lung cancer progression (Park et al., 2024). Hematopoietic aging promotes cancer by fueling IL-1α-driven emergency myelopoiesis and increased accumulation of myeloid progenitor-like cells in lung tumors (Park et al., 2024).

## Alzheimer’s disease

AD is a chronic aging-associated neurodegenerative disease and is the most common cause of dementia (Krishnamurthy et al., 2025). Certain cellular changes observed in AD are similar to those observed during the senescence of brain cells (Behfar et al., 2022). High levels of SASP proteins, including IL-6, IGFBP, TGF-β, and MMP-10, have been reported in CSF and plasma from AD patients. Microglia-associated neuroinflammation (Ramaswamy et al., 2019) persists throughout the neurodegenerative process, yet phagocytosis of beta-amyloid (Aβ) decreases in AD (Fulop et al., 2016). Aged microglia can gradually adopt a senescent-like and dystrophic phenotype that also reduces their ability to clear Aβ (Bussian et al., 2018). Oxidative stress increases the number of senescent microglia in the brain, and the resulting increase in dysfunctional immune cells can result in increased Aβ generation, hyperphosphorylated-tau aggregation spread, proinflammatory cytokine release, synaptic loss, paracrine senescence, and inflammation (Kadowaki et al., 2005; Gorgoulis et al., 2019). Senescent microglia are associated with AD, with Aβ detected inside the microglia in brain slices (Keren-Shaul et al., 2017). Increased pro-inflammatory cytokine expression with aging induces chronic systemic “inflammation” that increases over time, increasing RONS levels, cellular injury, and causing early death. Previous reports have shown that dystrophic and senescent microglia are observed in AD pathogenesis (Streit et al., 2009). Senescent cells increase in the brain tissue of AD patients and AD mouse models. The targeted removal of senescent cells in AD mice has shown beneficial effects by reducing protein aggregation, neurodegeneration and cognitive decline, offering a promising new therapeutic target for AD. Consequently, drugs that selectively eliminate senescent cells are currently under evaluation in clinical trials as potential therapies for AD patients (Ma et al., 2025). However, studies have suggested that chronic neuroinflammation mediated by activated microglia can cause neurofibrillary degeneration, and anti-inflammatory drugs have not been shown to suppress or reverse neuronal tau pathology (Streit et al., 2009).

Senescent astrocytes are reported to decrease physiological function and increase secretion of senescence-associated SASP factors, which lead to Aβ accumulation, tau hyperphosphorylation, neurofibrillary tangles (NFTs) formation, and cognitive dysfunction in AD (Han et al., 2020). Both Aβ and hyperphosphorylated tau can induce astrocyte senescence with a SASP profile, which causes the release of proinflammatory cytokines and causes cell death (Han et al., 2020). Senescent astrocytes induce glutamate cytotoxicity, synaptic dysfunction, loss of neuronal stem cells, and BBB disorders (Han et al., 2020). Senescent astrocytes have also been demonstrated to display upregulated p16INK4a expression, β-galactosidase activity, and SASP in cultures from post-mortem AD brain tissue (Behfar et al., 2022). These senescent astrocytes are abundant in both aging and neurodegenerative diseases. The Fragile X protein (FXP) family (FMR1, FXR1 and FXR2) is differentially expressed in major neurodegenerative diseases (Menge et al., 2025). Reduced expression of FXPs in neurodegeneration can contribute to pathogenic protein aggregation and death of vulnerable neurons in neurodegenerative disorders (Menge et al., 2025).

## Parkinson’s disease

PD is an age-related neurodegenerative disease that mainly affects older adults, with the risk of developing PD dramatically increasing after age 55 years, suggesting that immunosenescence may be a particularly relevant risk factor for disease development (Chaudhary et al., 2025; Ma et al., 2025). PD has different causes between younger and older people and shows differences in the progression of disease severity and pathologies according to age of disease onset, with young patients having less cognitive impairment and more motor dysfunctions (Di Lazzaro et al., 2025). Neuroinflammation is an important contributor to PD pathogenesis, along with the dual role of microglia in this disorder (Thangavel et al., 2024; Kempuraj D et al., 2024; Kempuraj et al., 2019). Older individuals may exhibit a broader spectrum of disease mechanisms, including increased prevalence of amyloid pathology and neurodegeneration (Di Lazzaro et al., 2025). Young patients had low levels of inflammatory mediators (YKL-40) and degeneration biomarkers (tau species, neurofilament light and heavy chains), which are independent of disease duration (Di Lazzaro et al., 2025). The number of CD8^+^ TEMRA T cells is decreased in PD patients compared to controls and the expression of p16^INK4a^ in CD8^+^ lymphocytes is also reduced in PD patients. Chronic latent cytomegalovirus (CMV) infection has been associated with elevated senescent CD8^+^ T cells (Kouli et al., 2021). Analyses of telomere length in PD subjects have been inconsistent, and it remains unclear whether cellular senescence is an initiator or driver of PD neuropathology. Existing research is still relatively less, and further studies are needed to assess the complex relationship between PD neuropathogenesis and cellular senescence (Ma et al., 2025). Potential for an innovative approach exists, as selective reduction of senescent astrocytes can mitigate nigrostriatal degeneration in mouse models of PD (Chen et al., 2024). Evidence supports senescence as a contributor to neuroinflammation in PD, but whether it primarily acts as an amplifier or an initiator remains unresolved. Panels covering SASP and glial-state markers could help clarify these roles in early cohorts (Russo and Riessland, 2022).

Peripheral blood mononuclear cells (PBMCs) are also useful for detecting cellular senescence in individuals with AD, mild cognitive impairment (MCI), PD, MS, and amyotrophic lateral sclerosis (ALS) (Pansarasa et al., 2022; Salech et al., 2022) (Table 1). Research has shown that specific markers of cellular stress and inflammation sourced from PBMCs can be used to potentially demonstrate worsening neurological symptoms or cognitive decline, hence providing valuable insight into disease mechanisms, progression, and treatment response (SanMartin et al., 2023; Tan et al., 2007).

## Viral reactivation and neurodegenerative diseases

The reactivation of latent viruses in the brain, i.e., herpes simplex virus (HSV), might initiate chronic neuroinflammatory reactions, mostly through activating microglia and astrocytes, leading to the expression of inflammatory mediators such as IL-1β. This inflammatory response drives synaptic dysfunction, cognitive deficits, and neuronal loss (Chauhan et al., 2025; Leblanc and Vorberg, 2022). Aβ accumulation and tau hyperphosphorylation in AD are associated with the HSV-1 reactivation (D'Aiuto et al., 2025; De Chiara et al., 2019; Leblanc and Vorberg, 2022). Moreover, oxidative stress is thought to be consequential after viral reactivation (Kayesh et al., 2025). Interestingly, physical or psychological stress can induce viral reactivation, which might amplify neurodegenerative processes in vulnerable individuals (Cairns et al., 2025). EBV reactivation has been strongly linked to Multiple Sclerosis (MS), a chronic inflammatory demyelinating disease. Immune cell populations from CSF and peripheral blood are altered, possibly due to viral reactivation in MS (Gouzouasis et al., 2025). Another study reported the presence of EBV latent proteins in B-cell follicles of MS postmortem brains (Serafini et al., 2007). In addition to EBV, reactivation of HHV-6 has also been reported in MS. Thus, an increased antibody response to viral antigens correlates with disease severity and immune activation, including cytokine dysregulation (Almulla et al., 2025).

## Myalgic encephalomyelitis/chronic fatigue syndrome (ME/CFS)

ME/CFS is a disabling, complex, chronic multisymptomatic disease associated with fatigue and neurological symptoms, including sleep disturbance, cognitive impairment, dysautonomias and persistent fatigue (Cohen et al., 2024; Gamer et al., 2025; Rajeevan et al., 2018). Major risk factors and symptoms of ME/CFS include premature telomere attrition and immune and cognitive dysfunction, which are similar to other conditions associated with accelerated aging (Rajeevan et al., 2018; Eaton-Fitch et al., 2024; Bansal et al., 2025). Viral reactivation, such as Epstein-Barr virus (EBV) and human herpesvirus 6 (HHV6), as well as persistent low-grade systemic inflammation, could play an important role in the pathogenesis of ME/CFS (Bansal et al., 2025; Vojdani et al., 2023; Cohen et al., 2024). Both ME/CFS and long COVID share many characteristics, such as fatigue, cognitive impairment, autoantibodies against neurons, endothelial disorder, mitochondrial abnormality, chronic neuroinflammation, and a pro-inflammatory gut microbiome (Komaroff and Dantzer, 2025; Tziastoudi et al., 2022). Aging, inflammaging, neurosenescence, and immune dysfunction could accelerate the onset and amplify the disease severity of ME/CFS (Luo et al., 2025).

Biomarkers for translation include Composite SASP profiles in CSF/plasma, along with BBB-leak indices and glial-state markers provide practical and pharmacodynamic endpoints and may also support enrichment of senescence-associated subgroups in AD and PD.

## Senolytics

Since an accumulation of senescent cells can damage surrounding tissue through the secretion of inflammatory SASP factors, compounds that either remove senescent cells or suppress the SASP may therefore offer therapeutic benefits to age-related conditions (Ma et al., 2025). The development of senolytic drugs has shown that targeting senescent cells is a valid method to treating age-associated diseases or slowing the progression of patients’ conditions (Lelarge et al., 2024). Senolytics are therapeutic agents that selectively eliminate senescent cells and have been shown to reduce cognitive decline in mouse neurodegenerative disease models, possibly by reducing SASP cytokine output, and reducing tau accumulation, while improving motor function and cognition (Bussian et al., 2018). Plant-derived flavonoids have been suggested to reduce age-associated skin appearance by targeting the key senescence pathway, SASP (Domaszewska-Szostek et al., 2021) and they are also anti-inflammatory and neuroprotective (Tsilioni et al., 2025; Kempuraj et al., 2021; Theoharides and Tsilioni, 2018). Several targets, such as BCL-2 proteins, tyrosine kinase/BCL-2 family, antioxidant, HSP90 inhibitors, p53, NA+/K+ ATPase and galactosidase prodrugs, are reported to be useful for treating age-related and other diseases by reducing senescent cells and SASP factors (Lelarge et al., 2024). In addition to senolytic therapies, several nonlytic compounds have shown the ability to eliminate senescent cells (Behfar et al., 2022). It has been suggested that blocking the programmed cell death protein-1/programmed death ligand 1 (PD-1/PD-L1) signaling could be a potential anti-aging senolytic therapy (Salminen, 2024). Senolytics may also be used as a co-treatment with other therapeutics if senolytic monotherapy does not provide sufficient therapeutic benefits. The PD-1/PD-L1 axis has been suggested to enhance the aging process. Overall, senolytic interventions may be useful for the treatment of neurodegenerative disorders (Jaganathan et al., 2025). Recently, the polyamine spermine has been shown to reduce the aging activity (Yang et al., 2025; Arthur et al., 2024; Uemura et al., 2023). Interestingly, spermine was reported to inhibit mast cells (Vliagoftis et al., 1992).

## Computational modeling for neuroinflammatory and neurodegenerative disorders

**FIGURE 2:**
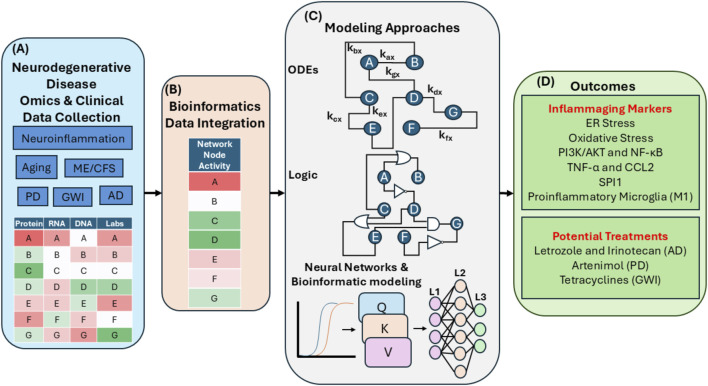
An illustrative example of computational modeling that links neurodegenerative diseases to inflammaging markers and treatments. **(A)** Multi-omics patient data are extracted from proteomics, transcriptomics, whole-genome sequencing, and clinical laboratory measurements for any of the listed neurodegenerative diseases. **(B)** The multi-omics data are integrated into a representative state for nodes within a given biological network using bioinformatics-based normalization approaches. **(C)** This integrated dataset is then used to represent the biological network with several modeling techniques. At the top of **(C)**, ODE-based modeling is depicted with nodes A-G representing key cellular genes, proteins, or RNAs. Kinetic rates are denoted as K_a-g_(x) to characterize network dynamics. Logic-based modeling is depicted in the middle of **(C)**. Connections between biological nodes are represented using logic gates (“AND”, “OR”, and “NOT”). The state of the nodes depends on these logic gates for processing key network regulators. At the bottom of **(C)**, a neural network-based approach is shown. Additional bioinformatic transformations are used to prepare the data as input for the neural network. The data are then fed through a modern neural network architecture that includes the core components of the transformer attention mechanism, the Q, K, and V matrices, as well as a connected layer of artificial neurons. The neural network then extracts critical features from the biological network. **(D)** Outcomes of modeling approaches in neurodegenerative disease and inflammaging are summarized, highlighting key inflammaging markers and potential therapies identified across different neurodegenerative diseases. AD, Alzheimer’s Disease; AKT, AKT Serine/Threonine Kinase; CCL2, C-C Motif Chemokine Ligand 2; ER, Endoplasmic Reticulum; GWI, Gulf War Illness; K, Key Matrix; ODEs, Ordinary Differential Equations; ME/CFS, Myalgic encephalomyelitis/chronic fatigue syndrome; PI3K, Phosphatidylinositol-3 Kinase; PD, Parkinson’s Disease; Q, Query Matrix; SPI1, Spi-1 Proto-Oncogene; TNF-α, Tumor Necrosis Factor α; V, Value Matrix.

Advances in computational modeling work have tied neurodegenerative diseases to neuroinflammation and cellular senescence signaling cascades (Figure 2). Modeling techniques used to probe neurodegenerative diseases include ordinary differential equations (ODE)-based modeling, logic modeling, and neural network-based modeling. ODE-based modeling assigns kinetic rates to known interactions in a biological network (Hasdemir et al., 2015). This approach requires setting specific rates for each interaction, which can be challenging for complex networks. For example, in PD research, one approach described how dopamine and α-synuclein interact to trigger apoptosis through the enhancement of ROS production (Buchel et al., 2013). Similarly, in AD, an ODE-based model described how Aβ promotes a cascade activating microglia; first, anti-inflammatory microglia (M2) are activated and then eventually overwhelmed by a proinflammatory microglial (M1) response driven by TNF-α and CCL2 (Chamberland et al., 2024). This proinflammatory microglial activation is also observed in cellular senescence mechanisms and aging (Wendimu and Hooks, 2022).

Logic modeling is particularly useful when it may be difficult to assign kinetic rates to network interactions. In these instances, logic gates such as “AND”, “OR” and “NOT” gates are used to establish a set of rules to determine activation or inhibition (Schwab et al., 2020). Ternary logic schemes describe biological networks as “on” or “off” (Boolean), or “down,” “normal,” or “up” (ternary) (Gates et al., 2021; Craddock et al., 2014). Nodes within a network can be updated together (synchronously), one by one (asynchronously), or probabilistically (Schwab et al., 2020). This approach can uncover biomarkers and therapy suggestions. For example, ternary logic-based simulations investigating GWI determined that broad-acting anti-inflammatory agents, tetracyclines, have promise in controlling neuroinflammation. However, certain anti-inflammatory drugs such as propranolol (β2-adrenoreceptor antagonist) have been shown to increase the expression of α-synuclein gene (SNCA) in the neurons and increase the risk for PD (Mittal et al., 2017; Nguyen et al., 2025). In PD research, combining logic modeling with early and prodromal PD patient omics detected changes in oxidative stress, autophagy, mitochondrial fission/fusion, and mitochondrial biogenesis (Hemedan et al., 2024). These changes are all hallmarks of inflammaging (Jurcau et al., 2024). Likewise, combining single-cell omics data and PD-based logic maps found novel points of disease intervention, including SEC63 & KDELR1. This approach also implicated the known inflammation-related pathways PI3K/AKT and NF-κB (Mihajlovic et al., 2024). Additional analysis uncovered repurposed treatments to target these pathways, such as Artenimol (Mihajlovic et al., 2024). Comparable approaches have been employed to uncover potential subgroups of AD (Choi et al., 2024). AD was divided into 9 separate subtypes utilizing genomics data and logic modeling; three key genes were used to stratify the cohort: SPI1, CASS4, and MEF2C. SPI1 is an inflammatory regulator that is linked to proinflammatory microglial signaling in AD (Kim et al., 2024).

Recent advances in neural network performance in several areas have led to its increased use in the computational modeling of inflammatory disease pathology. Neural network-based modeling involves constructing a network of artificial neurons and then feeding feature-rich data to adjust the connection strength between neurons. This process allows the model to predict, design or categorize key features of similar input data (Sarker, 2021). The discovery and design of mechanisms of “attention” and the transformer architecture have catapulted the field of neural network modeling forward (Yin et al., 2024; Choi and Lee, 2023). These breakthroughs have led to advances in natural language processing, drug design, and multi-omics processing (Yin et al., 2024; Zhang et al., 2023; Choi and Lee, 2023). In the case of biological networks, combined transformer architecture and a panel of inflammatory markers predicts simulated age for control patients and end-stage renal disease patients. This study identified how simulated age is accelerated in end-stage renal disease patients (Kalyakulina et al., 2023). CXCL9, CCL22, and IL-6 were the top inflammatory markers driving simulated age prediction, though this approach has not yet been directly applied to simulate brain age. In another neural network-based approach aimed at uncovering therapies for AD, single-cell omics data, machine learning, and bioinformatics-based statistical approaches were employed to uncover the therapeutic combination of letrozole and irinotecan; these drugs were found to reduce astrocytic and microglial inflammatory signaling in an animal model (Li et al., 2025). Interestingly, the proinflammatory microglial markers TNF-α and CCL2 were identified as potential biomarkers from blood and extracellular vesicles from ME/CFS patients; TNF-α and other proinflammatory markers predicted ME/CFS with 86.1% accuracy (Giloteaux et al., 2023). CCL2 was associated with increased aging only in ME/CFS patients but not in controls. Digital twin technology, which includes computational models that represent patient health states over time, is another promising technological tool for disease modeling. Important immune features can be implemented into medical digital twins to improve their clinical use and accuracy (Niarakis et al., 2024). Overall, diverse modeling approaches have uncovered markers linking neurodegenerative diseases to inflammaging in age-associated diseases and have helped to guide potential treatments (Figure 2).

## Conclusions and future perspective

Aging is a normal biological process that causes the steady deterioration of cellular structures, resulting in a progressive impairment of function, which is termed senescence. Although senescence plays an essential role in development, tissue homeostasis and tumor suppression, it is also associated with many age-related diseases. Aging causes thymic involution, reduced telomerase activity, decreased immune response to vaccination, increased susceptibility to infection, heightened inflammatory reactions and autoimmunity, and an observed decline in homeostatic mechanisms. Immunosenescence has been reported in many chronic age-related inflammatory diseases. Inflammaging contributes to elevated inflammatory mediators and tissue dysfunction. During aging, the pools of naïve T and B cells are reduced, with elevated CD8^+^ and a reduction in CD4^+^ T cells. Cellular aging leads to cell cycle arrest in response to stress and neuronal injury, and this halting of proliferation further decreases the tissue’s proportion of healthy cells. Neurodegenerative diseases are linked to cellular senescence caused by oxidative stress, ROS generation, and DNA damage. Consequently, targeting cellular senescence could have therapeutic potential for treating neurodegenerative diseases (Jaganathan et al., 2025). Senolytic therapy also has the unique ability to alleviate physiological aging of the human brain as well, even when linked with the pathology of other infectious diseases such as COVID-19 (Aguado et al., 2023). Recent studies indicate that genetic or pharmacological removal of senescent cells increases life span and improves quality of life (Mohapatra et al., 2023). Additionally, reports also suggest that cell-cycle arrest is not always a defining feature of senescence, as post-mitotic cells, already unable to proliferate, can become senescent, and some senescent cells can re-enter the cell cycle (Gorgoulis et al., 2019). Future studies should focus on the contribution of age-related microbial shifts, lifestyle stressors, and metabolic alterations, all of which accelerate immune aging and may influence vulnerability to neurodegenerative and inflammatory diseases. The important environmental factors and toxicants should also be studied on-a-chip to mimic aging processes in the brain models and could provide *in vitro* models for investigating therapeutic agents (Yang et al., 2025; Chen et al., 2025; Kamroo et al., 2025; Fagiani et al., 2025). The influence of psychological factors on the immune system in young and aged, as well as the role of gut microbiota in immunosenescence, warrants further in-depth research.
